# Validity of questions about activities of daily living to screen for dependency in older adults

**DOI:** 10.11606/S1518-8787.2017051006959

**Published:** 2017-08-25

**Authors:** Monica Rebouças, João Macedo Coelho-Filho, Renato Peixoto Veras, Maria Fernanda Lima-Costa, Luiz Roberto Ramos

**Affiliations:** I Programa de Pós-Graduação em Saúde Coletiva. Escola Paulista de Medicina. Universidade Federal de São Paulo. São Paulo, SP, Brasil; II Departamento de Medicina Clínica. Faculdade de Medicina. Universidade Federal do Ceará. Fortaleza, CE, Brasil; III Universidade Aberta da Terceira Idade. Universidade do Estado do Rio de Janeiro. Rio de Janeiro, RJ, Brasil; IV Centro de Pesquisa René Rachou. Fundação Oswaldo Cruz. Universidade Federal de Minas Gerais. Belo Horizonte, MG, Brasil; V Departamento de Medicina Preventiva. Escola Paulista de Medicina. Universidade Federal de São Paulo. São Paulo, SP, Brasil

**Keywords:** Aged, Activities of Daily Living, Independent Living, Diagnostic Self Evaluation, Surveys and Questionnaires, utilization, Validation Studies

## Abstract

**OBJECTIVE:**

To determine the criterion validity of the activities of daily living present in functionality questionnaires in older adults for population surveys and to identify which activities are valid to quantify the real daily need for help of this population.

**METHODS:**

This is a population sample of older adults stratified by levels of functionality, according to self-perception of dependency in the activities of daily living. Self-perception was compared with the gold standard – direct observation of these activities in the household of older adults by a trained professional, blinded to the answers in the questionnaire. At the visit, it was decided if the older adult needed help to perform any of the activities of daily living for the research. The sensitivity of each activity of daily living was greater when the self-assessment that there was no need for help coincided with the assessment of the professional. Specificity indicates coincidence regarding the need for help in the activities of daily living – coefficients of sensitivity and specificity above 70% were considered as indicative of good validity.

**RESULTS:**

Self-assessments showed better sensitivity than specificity – older adults and observers agreed more on daily independency than on dependency. All activities showed sensitivity above 70%. Some activities had low (go shopping: 55%) or very low specificity (brush the hair: 33%). The best specificities were to take a shower and dress up (95.8% for both), among the personal ones, and to use transportation and perform banking transactions (78% for both), among the instrumental ones.

**CONCLUSIONS:**

Activities of daily living can be valid indicators of functional dependence. The best coefficients of validity were generally obtained for personal activities. Some activities with good sensitivities and specificities – walk 100 meters, take a shower, and lie down in and get out of the bed – can be used to classify older adults into low, average, and high need for help depending on the affected activities and, therefore, can help in the planning of health services aimed at them.

## RESUMO

**OBJETIVO:**

Determinar a validade de critério das atividades de vida diária constantes em questionários de funcionalidade em idosos para inquéritos populacionais e identificar quais atividades são válidas para quantificar a necessidade real de ajuda diária dessa população.

**MÉTODOS:**

Amostra populacional de idosos estratificada por níveis de funcionalidade, segundo a autopercepção de dependência nas atividades de vida diária. Autopercepção foi comparada com o padrão ouro – observação direta no domicílio dos idosos nessas atividades por profissional treinado e cego para respostas no questionário. Na visita decidiu-se, para efeito da pesquisa, se o idoso precisava de ajuda para realizar alguma das atividades de vida diária. A sensibilidade de cada atividade de vida diária foi maior quando a autoavaliação de que não havia necessidade de ajuda coincidiu com a avaliação do profissional. A especificidade indica coincidência quanto à necessidade de ajuda na atividades de vida diária – coeficientes de sensibilidade e especificidade acima de 70% foram considerados indicativos de boa validade.

**RESULTADOS:**

As autoavaliações mostraram sensibilidade melhor que especificidade – idosos e observadores concordaram mais quanto à independência do que quanto à dependência cotidiana. Todas as atividades mostraram sensibilidade acima de 70%. Algumas atividades tiveram uma especificidade baixa (fazer compras: 55%) ou muito baixa (pentear os cabelos: 33%). As melhores especificidades foram tomar banho e vestir-se (95,8% ambas), entre as pessoais, e utilizar transporte e realizar movimento bancário (ambas com 78%), entre as instrumentais.

**CONCLUSÕES:**

Atividades de vida diária podem ser indicadores válidos de dependência funcional. Os melhores coeficientes de validade em geral foram obtidos pelas atividades pessoais. Destacam-se algumas atividades com boas sensibilidades e especificidades – andar 100 metros, tomar banho e levantar/deitar na cama – que podem servir para classificar os idosos em baixa, média e alta necessidade de ajuda dependendo das atividades afetadas e, dessa forma, auxiliar no planejamento dos serviços de saúde voltados aos idosos.

## INTRODUCTION

In the scenario of population aging in Brazil – an exponential increase in the elderly population and consequently the burden of potentially incapacitating chronic noncommunicable diseases (NCD) –, life expectancy is analyzed taking into account not only survival, but also disability-adjusted life years (DALYS)^25^. The contemporary concept of health considers as healthy the individual with full functional capacity, even if diagnosed with multiple NCD. This concept privileges the daily independency and autonomy of the individual as fundamental variables^29^.

Currently, the degree of incapacitation, dependency, and need for help of the users of the health system should be considered in the planning of public health actions. To this end, simple, valid, and reliable indicators are needed to identify and quantify daily dependency and the need for help^30^.

Most of the instruments used to assess the functional capacity of older adults in population surveys in Brazil are multidimensional and include functional scales based on the subjective perception of self-competence to perform activities of daily living (ADL)^2^ ^,^ ^15^ ^,^ ^27^ ^,^ ^34^. They are questions about the need for help to carry out personal care activities and instrumental activities in society, such as to take a shower, go shopping, or clean the house. They are questionnaires based on scales that aim to assess competence in self-care after hospital discharge, or in the follow-up of chronic patients, or to assess the ability to maintain independent living in society^18^ ^,^ ^20^ ^,^ ^22^ ^,^ ^28^.

Although these different questionnaires and scales are widely used in research, there are few studies on the validity of these instruments to quantify the prevalence of dependency in a population^4^ ^,^ ^14^ ^,^ ^17^ ^,^ ^19^ ^,^ ^26^. Important bias may exist between self-assessment of dependency and the assessment done by a health professional, which can, for example, evoke new metrics to assess competence in ADL^11^ ^,^ ^30^. Particularly in Brazil, there are no studies that have investigated the validity of the questions about ADL to indicate the dependency of the respondent.

In 2013, a multicenter population-based study on functionality investigated the questions about ADL in use in the country, identifying three constructs underlying the subject of daily functionality in older adults: 1) personal activities at home that necessarily involve the older adult (e.g., take a shower), 2) household activities that others can do for the older adult (e.g., wash clothes), and 3) activities that require mobility outside the home (e.g., go shopping). The findings reveal that questions about daily acts overlap in different constructs, but some activities are better indicators of function than others, within each construct^30^.

In this context, this study aimed to examine the validity of each question about the need for help in ADL to detect daily dependency. The self-perception of dependency in all activities present in the main instruments of functional assessment in use in Brazil was compared to a gold standard based on direct observation by a trained professional of the performance of the individual in the activities.

## METHODS

Between 2007 and 2009, a multicenter national household survey was carried out with approximately 5,000 older adults who answered a questionnaire about the need for help in ADL in four Brazilian cities (São Paulo, Fortaleza, Rio de Janeiro, and Bambuí)^30^. For this study, we used data from São Paulo and Fortaleza. The questionnaire contained 20 ADL compiled from existing instruments in use in the country – BOMFAQ^27^, BOAS^34^, SMAF^15^, and MIF^2^, being them: go shopping, use transportation, perform banking transactions, handle day-to-day money, control feces or urine, go up the stairs, walk 1 km, walk 100 meters, walk at home on flat surfaces, prepare a meal, wash clothes, dress up, take a shower, eat a meal, use the telephone, wear or remove prostheses, cut toenails, brush the hair, sit or stand up from the chair, and lie down in or get out of the bed. Although extracted from different instruments, the questions about all the ADL selected had common wording for this study^30^.

The strength of this validation study is that the sample of older adults was randomly selected from a population sample, previously interviewed at home, which can be stratified according to the degree of functionality. There was no exclusion criterion, since the objective of the study was to assess the criterion validity of each of these activities to detect the need for help among the older adults in the general population. Based on the answers given regarding the need for help in the survey, the older adults in Fortaleza and São Paulo were stratified according to the number of activities that required help in the self-assessment. Three extracts were created – one for low dependency (0–2), one for moderate dependency (3–9), and one for high dependency (10–20) – that were arbitrarily defined, since the 20 questions compiled did not correspond to any specific scale that had defined cut-off points. For each of the 40 questions (20 on the difficulty and 20 on the need for help), five older adults were selected. Of the 400 persons selected in the two cities (200 were drawn to make a sample reserve in cases of refusals), 30% were from the “dependent” strata, 30% from the “intermediary” strata, and 40% from the “independent” strata. The stratification intended to repeat in the sub-sample the expected prevalence in the community.

In the validation stage, presented here, older adults were visited by a team of health professionals (four physiotherapists and two physicians with some training in gerontology) trained to assess the functionality of the older adult with similar criteria. The observers always worked in pairs, with one applying the performance script in the activities while the other only observed, being both blinded to the previous self-assessment of the older adult. The script contained steps that the examiner should follow to verify the independence of the older adult when performing the activities in the questionnaire, such as “ask him or her to remove and use the denture, hearing aid, or other prosthesis if the elderly person uses them”, or “ask the older adult to remove three pieces of clothing and wear them, including shoes; the steps are: putting on clothes, buttoning them, closing or opening the zipper, and tying or closing the shoe”. Some activities were tested with the help of a kit with fake money, plastic nail clippers, and a machine to calculate expenses. The pairs alternated the roles of conductor and observer of the script. Verbal contact outside of the script was discouraged; only the inevitable was allowed when visiting the household. The script followed the steps suggested by Minayo^24^ for a directed observation and it was created especially for the study presented here to assess independence in the ADL of older adults.

Kappa and Spearman coefficients were calculated to assess the agreement of all pairs in each activity (interobserver reliability). Intraobserver reliability could be calculated for two observers who revisited the same older adult after approximately one week (data not shown, presented in electronic media attached to the thesis deposited in the thesis database of CAPES)^31^.

In the end, observers had to decide, for the research, whether the older adult needed help from third parties to perform the activities or not. This condition of dependency, assumed as a gold standard, was analyzed according to the answers “yes, I need help to...” for each of the ADL in the questionnaire, which allowed the calculation of the validity (sensitivity and specificity) of each ADL studied to identify the older adult who needs daily help.

In this study, the sensitivity values of each ADL define the extent to which the older adult self-assessment as independent in that ADL (without need for help) coincides with the professional’s assessment. Specificity identifies the older adult who claims to “need help for an ADL” and coincides with the assessment of the gold standard. Low sensitivities and specificities reflect situations in which older adults and professionals disagreed regarding independency and dependency, respectively. Coefficients of sensitivity and specificity above 70% were considered as good.

The older adults signed an informed consent and the research was approved by the Ethics Committees of the two universities involved (Universidade Federal de São Paulo – UNIFESP 0574/09; Universidade Federal do Ceará – UFC 155/08).

## RESULTS

In general, the self-assessments of the older adults about the need for help to perform each activity showed better sensitivity than specificity, that is, older adults and health professionals agree more on daily independency than on dependency.

Approximately 202 older adults were included in the study (100 in Fortaleza, State of Ceará, and 102 in São Paulo, State of São Paulo). In general, 59% were women (mean age of 75.5 years) and 41% were men (mean age of 74.2 years). Approximately 20% of the older adults included had reported severe dependency in the household survey (22% in São Paulo and 20% in Ceará).

The measures of reproducibility estimated varied between the pairs of observers and between the activities observed. Values above 0.4 for Kappa (K) and 0.75 for Spearman (S) were considered as good. The activities that presented the best reproducibility in the average of observations in São Paulo and Fortaleza were: take a shower (K = 82.2; S = 0.92), dress up (K = 68.4; S = 0.83), walk on a flat surface (K = 63.8; S = 0.71), and lie down in/get out of the bed (K = 66.8; S = 0.80) (data not shown in table).

In the total sample, all activities showed sensitivity above 70%, while seven activities had low specificity (go shopping: 55%, handle money: 64%, walk 1 km: 50%, walk on a flat surface: 67%) or very low specificity (eat a meal: 46%, use dentures: 33%, brush the hair: 33%). In São Paulo, only two ADL showed low sensitivity (prepare a meal [64%] and wash clothes [69%]), while five ADL had specificity below 70% (handle money: 58%, control sphincters: 53%, walk 1 km: 37%, walk on a flat surface: 54%, use dentures: 33%). In Fortaleza, all ADL showed good sensitivity, but seven ADL had low specificity (go shopping: 55%, handle money: 64%, walk 1 km: 50%, walk on a flat surface: 68%, eat a meal: 46%, use dentures: 33%, brush the hair: 33%). Older adults who report dependency confirmed by a health professional were more frequent in São Paulo than in Fortaleza (Table).

**Table:** Estimates of sensitivity (Sens) and specificity (Spec) of the questions about activities of daily living regarding levels of need for help of older adults, in São Paulo (SP), Ceará (CE), and São Paulo and Ceará (SPC), 2009.

Activity	Sens* SP %	Spec* SP %	Sens* CE %	Spec* CE %	Sens* SPC %	Spec* SPC %
Go shopping	68.8	**100**	**86.3**	55.0	**77.5**	70.0
Use transportation	**77.2**	**95.0**	**85.7**	65.5	**81.2**	**77.6**
Perform banking transactions	**81.2**	**93.8**	**89.1**	**73.6**	**84.3**	**78.3**
Handle day-to-day money	**98.7**	58.3	**91.0**	63.6	**94.6**	60.9
Control feces and urine	**98.8**	52.9	**94.8**	**100**	**96.7**	60.0
Go up the stairs	**94.7**	**79.2**	**86.5**	**79.2**	**90.7**	**79.2**
Walk a distance of 1 km	**100**	36.7	**97.0**	50.0	**98.0**	41.8
Walk a distance of 100 m	**87.3**	70.0	**97.4**	**90.0**	**92.3**	**80.0**
Walk around the house, on flat surfaces	**98.9**	53.8	**96.7**	66.7	**97.8**	59.1
Cook a meal	64.1	**83.3**	**94.5**	**77.3**	**78.8**	**78.6**
Wash clothes	69.4	**100**	**88.2**	70.0	**79.1**	**72.7**
Dress up	**89.4**	**100**	**98.9**	**85.7**	**94.3**	**95.8**
Take a shower	**96.4**	**94.4**	**100**	**100**	**98.3**	**95.8**
Eat a meal	**91.4**	**100**	**100**	46.2	**95.5**	68.2
Use the telephone	**90.2**	**77.8**	**94.9**	**75.0**	**92.5**	**76.5**
Wear and remove prostheses	**96.9**	33.3	**98.6**	33.3	**97.8**	33.3
Cut toenails	**77.9**	**97.0**	**88.1**	**80.0**	**83.0**	**88.9**
Brush the hair	**95.9**	**80.0**	**96.8**	33.3	**96.3**	54.5
Sit down and get up from the chair	**93.4**	**81.8**	**97.8**	**85.7**	**95.6**	**83.3**
Lie down in and get out of the bed	**94.6**	**100**	**96.7**	**83.3**	**95.7**	**93.8**

* Coefficients of Sensitivity and Specificity above 70% were considered as good and highlighted in bold.

In general, sensitivity and specificity values were higher for questions about basic activities than for questions about instrumental activities, suggesting greater disagreement between older adults and health professionals about the concept of dependency in complex activities. The ADL with better specificities were generally obtained by asking about activities such as “take a shower” and “dress up” (95.8% for both), and lie down in/get out of the bed (94%). Among the instrumental ADL, those with the best specificity were: use transportation and perform banking transactions (78% for both) and prepare a meal (79%). Go up the stairs and walk 100 meters also showed good specificity (79% and 80%, respectively) (Table).

## DISCUSSION

The study suggests, based on the results, that ADL may be valid indicators of functionality. The best coefficients of validity were obtained from the questions about the need for daily help to: take a shower, dress up, lie down in and get out of bed, sit down and get up from the chair, walk a distance of 100 meters, go up the stairs, and use transportation.

Since the 1960s, there have been studies on scales to define functional capacity. The Lawton and Brody scale, for example, has been tested by comparison with another scale, the PSMS – Physical Self-maintenance Scale^19^. The SIP – Sickness Impact Profile –*,* of Bergner et al., has been validated by comparison with the opinion of physicians and with instruments, such as Katz^3^ ^,^ ^4^. Brorsson and Asberg, in Switzerland, have published work on the reliability and validity of the ADL Index or Katz Index, from the observation of nurses in institutionalized older adults^5^. The reproducibility of the Barthel scale shows low Kappa^35^ and validity values; comparing the results with the Motor Scale, it presents good correlation^1^. The publication of Fillenbaum and Smyer on the reproducibility and validation of Older Americans Resources and Services (OARS) multidimensional functional assessment questionnaire exposes the controversies about test measures performed with ADL questionnaires, as the questions presented very poor reproducibility, which leads to the low agreement between observers and respondents in the opinion of the authors^10^. The same OARS presents a high correlation when compared to the results of the Functional Autonomy Measurement System (SMAF)^9^ ^,^ ^15^ ^,^ ^23^.

We emphasize that different studies in the literature have defined validation as the agreement between questionnaires without the use of a gold standard^32^. Other authors have used as a gold standard the medical examination of patients, or the assessment of nurses in long-term institutions for older adults, which are recognized different populations^32^. In fact, there is a gap in the knowledge about the construct validity of the questions about ADL to indicate dependency, including internationally.

We used a gold standard based on the participative observation of a trained health professional, an unprecedented experience in Brazil, which is parallel to the method previously used in a home study in England^17^. A script of direct observation of trained professionals was elaborated about several daily actions performed in a simulated way by the older adult at home. We did not find in the literature an alternative gold standard for the comparison of a scale of ADL if not with other functional scales and measures, which also lacked criterion validation as indicators of daily need for help. In fact, all studies that use scales to estimate the functionality of an older population using scores from the number of impaired activities requiring help provide considerations about the limitations of using some specific activities (e.g., need for help to wash clothes in men who have never washed clothes, need for help to take care of finances in women who have never had this responsibility), which knowingly have biases and can inflate the score, but which are part of the scale and must be used to make the originally developed score. In practice, we have numerous scales, each with a different number of activities and scores, with low comparability, and criterion validity not studied.

Our objective was to estimate the sensitivity and specificity of each of the activities currently in use in Brazil, looking for those that seem to be better understood by interviewees and interviewers, resulting in a coincidence of assessments about the need for help or not – a better criterion validity. However, validation demands a gold standard. The direct observation has limitations, but we would be looking at the videomonitoring of the lives of these older adults in the extreme end so that self-assessment could be confronted with reality in an unmistakable way, which is not feasible in terms of research.

We trained qualified observers, who previously discussed the concepts and criteria about daily inability and dependency, to follow a common script in the home of the older adult, of whom they had no prior information. We constructed a possible gold standard with reasonable reliability that allowed us to quantify the validity of each ADL in use and thus provide simpler analysis logic. The results confirmed the low validity of some questions to identify a real need for help (e.g., wash clothes and prepare meals) and the high specificity of personal activities, such as take a shower and dress up.

Currently, the need to know more about the instruments of data collection for functionality is widely discussed, including their standardization in order to facilitate comparability between different countries and research studies^7^ ^,^ ^8^. Brazilian authors reinforce the criticism of the current model to assess functionality at the same time as initiatives such as SISAP-Aged, developed by the Ministry of Health, point to the use of functional indicators to guide strategies for the older population^16^ ^,^ ^21^.

Along these lines, our study meets the demand and adds instigating points. It innovates by presenting a population-based sample, which is random and from two distinct regions of the country, with information collected at home by direct observation, isolating activity by activity, and repeating the natural condition of life of most of the older adults in Brazil. Additionally, as a reinforcement of the quality of the study, masking was obeyed in relation to the answers of the questionnaire when the performance of the activities was observed.

A relevant point was the observation that the questions with the highest scores in the research repeat those suggested by Jefferys et al.^17^, in 1969, and reaffirmed by Guralnik et al.^15^, in 1994. These pioneer groups, with no followers, assessed questions about daily activities, comparing responses of older adults with physical ability tests. The mentioned studies highlight questions about physical fitness, which can be more objectively validated^7^ ^,^ ^8^. However, the same authors suggest, since the 1960s, the careful use of questions such as “walk” and “go up the stairs”, since they cover mobility, but not the broader functional dimensions^13^ ^,^ ^17^.

More recently, the study of Ramos et al.^30^ suggests, based on a factor analysis, that the ADL that best represent the dependency construct in older adults are “get out of the bed”, “take a shower”, and “walk 100 meters” (the first two are personal activities and the third is a mobility activity). These activities also revealed good coefficients of validity and reliability in our study. Personal activities, unlike more complex instrumental activities, have a clearer construct, which is reflected in better correlations when we compare the perception of the older adults with the opinion of health professionals.

All these studies confirm and complement each other, since they establish similar questions as the best indicators of functionality in the daily life of older adults using different strategies. It is worth emphasizing that activities with favorable values are repeated in different places and by different teams, thus suggesting that the relevant point is the subject of the question, and not its format. Therefore, we are talking about the capacity of generalization (or external validity) of the results presented here^14^ ^,^ ^17^ ^,^ ^30^.

The common way to assess functionality using “scales” mixes questions with good measurement properties with others with low validity coefficients. Although there is a hierarchy between the activities of the scales – the most complex are affected before the simplest –, the sum does not always reflect the reality of the degree of dependency of the individual^27^ ^,^ ^28^ ^,^ ^33^. In this study, we detailed each question and estimated their validity.

There were regional differences, with a trend of higher specificity in São Paulo. This region, because it is privileged in the provision of health services, can generate greater clarification about dependency. However, on daily independence, there was more agreement between professionals and older adults in Fortaleza, indicating possible cultural influence on the interpretation of the functionality in Brazilian older adults.

Two activities with good sensitivities and specificities stand out: “take a shower”, a basic, personal activity that involves the older adult, and “use transportation”, an instrumental activity that requires mobility outside the home. The performance of these activities without help presupposes independency, motivation, physical ability, and social engagement. The need for help to “use transportation” and “take a shower” is sentinel in depressive and dementia situations. Persons get lost in the streets when they have an impairment of mental function, and the premonitory symptoms are getting out of bed, getting dressed without help, but not wanting to “take a shower”^13^.

The findings of this study corroborate those of Spector et al.^32^, who highlight their role as indicators of dependency of the need for help to “use transportation” among the instrumental activities and “take a shower” among the personal ones. Similarly, Del Duca et al.^8^, 2009, emphasize “taking a shower and moving around using transportation” as indicators of problems among older adults.

When checking the properties of the questions in Brazilian Portuguese about the need for help in activities of daily living, we could conclude that, from some questions that alone have a good specificity to indicate the need for help – as for example, walk 100 meters, take a shower, lie down in/get out of bed – we can trace subgroups of older adults: the independent ones, who do not need help in any of these activities; those with mild dependency, who need help to leave the home and do activities outside (100 m); those with moderate dependency, who need frequent and in-home help to take a shower; and the completely dependent ones, who require help also for any movement in the house (getting out of bed). Future analyses may prove the predictive power of these questions in relation to existing scales and scores. The main objective will always be to increase the capacity of screening with instruments that are simpler to analyze and which have a good predictive power of the need for help. In the paradigm of population aging, the demand for care for older adults will increase greatly and this type of methodology may be vital for the planning and management of health services for older adults.
